# Computer-aided, resistance gene-guided genome mining for proteasome and HMG-CoA reductase inhibitors

**DOI:** 10.1093/jimb/kuad045

**Published:** 2023-12-07

**Authors:** Cory B Jenkinson, Adam R Podgorny, Cuncong Zhong, Berl R Oakley

**Affiliations:** Department of Molecular Biosciences, University of Kansas, Lawrence, KS 66045,USA; Department of Electrical Engineering and Computer Science, University of Kansas, Lawrence, KS 66045,USA; Department of Electrical Engineering and Computer Science, University of Kansas, Lawrence, KS 66045,USA; Department of Molecular Biosciences, University of Kansas, Lawrence, KS 66045,USA

**Keywords:** Secondary metabolism, Genome mining, Proteasome, HMG-CoA reductase, Resistance gene

## Abstract

Secondary metabolites (SMs) are biologically active small molecules, many of which are medically valuable. Fungal genomes contain vast numbers of SM biosynthetic gene clusters (BGCs) with unknown products, suggesting that huge numbers of valuable SMs remain to be discovered. It is challenging, however, to identify SM BGCs, among the millions present in fungi, that produce useful compounds. One solution is resistance gene-guided genome mining, which takes advantage of the fact that some BGCs contain a gene encoding a resistant version of the protein targeted by the compound produced by the BGC. The bioinformatic signature of such BGCs is that they contain an allele of an essential gene with no SM biosynthetic function, and there is a second allele elsewhere in the genome. We have developed a computer-assisted approach to resistance gene-guided genome mining that allows users to query large databases for BGCs that putatively make compounds that have targets of therapeutic interest. Working with the MycoCosm genome database, we have applied this approach to look for SM BGCs that target the proteasome β6 subunit, the target of the proteasome inhibitor fellutamide B, or HMG-CoA reductase, the target of cholesterol reducing therapeutics such as lovastatin. Our approach proved effective, finding known fellutamide and lovastatin BGCs as well as fellutamide- and lovastatin-related BGCs with variations in the SM genes that suggest they may produce structural variants of fellutamides and lovastatin. Gratifyingly, we also found BGCs that are not closely related to lovastatin BGCs but putatively produce novel HMG-CoA reductase inhibitors.

**One-Sentence Summary:**

A new computer-assisted approach to resistance gene-directed genome mining is reported along with its use to identify fungal biosynthetic gene clusters that putatively produce proteasome and HMG-CoA reductase inhibitors.

## Graphical Abstract

**Graphical Abstract:**
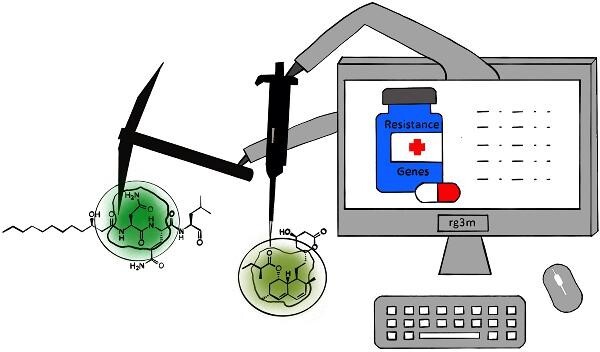

## Introduction

Fungi produce a plethora of biologically active small molecules, called secondary metabolites (SMs). Many fungal SMs are currently used in medicine, and they include antibiotics such as penicillin, antifungals such as the echinocandins, cholesterol-lowering agents such as lovastatin, and immune-modifying agents such as cyclosporine (reviewed in Bok et al., 2006; Demain, 2014; Keller et al., 2005; Martin, 1998; Palaez, 2005). Almost universally, the genes that encode particular SM biosynthetic pathways are clustered together in the genome, forming biosynthetic gene clusters (BGCs) (Galagan et al., 2005; Zhang et al., 2004). Secondary metabolite BGCs contain one or more core biosynthetic genes responsible for synthesizing the backbone of the SM as well as other genes that encode products that modify the backbone structure, influence production of the SM, or confer resistance to the SM. Generally, the genes within each BGC are coordinately regulated, in many cases being controlled by a transcription factor (TF) in the cluster (Brakhage, 2013; Keller et al., 2005; Keller, 2019; Macheleidt et al., 2016).

Secondary metabolite BGCs can be grouped into major classes based on their core biosynthetic genes and the building blocks they use for synthesis of the backbone structure. Non-ribosomal peptide synthetase (NRPS) BGCs utilize standard and modified amino acids; polyketide synthase (PKS) BGCs utilize acetyl-CoA and malonyl-CoA; terpene synthase and cyclase BGCs utilize isoprene units; and dimethylallyl tryptophan synthase (DMATS) BGCs use tryptophan and dimethylallyl pyrophosphate (Keller et al., 2005). These core biosynthetic backbone genes can be readily identified through bioinformatics due to their highly conserved structural domains (Andersen et al., 2013).

While the research community has discovered tens of thousands of fungal SMs over decades of research, genome sequencing and bioinformatics have revealed that the BGCs that make known SMs are only a small fraction of the total SM BGCs that exist. It follows that we have discovered only a fraction of the compounds that fungi are capable of creating (de Vries et al., 2017; Drott et al., 2020; Inglis et al., 2013; Lind et al., 2015; Nielsen et al., 2017). The fact that there are many more SM BGCs than known SMs reflects the fact that the majority of fungal SM BGCs are not expressed under the typical laboratory growth conditions that have been used for SM discovery. Perhaps more importantly, it illustrates that there is vast potential for the discovery of new bioactive molecules from fungi that can be used to improve human health.

Although most fungal SM BGCs are normally silent, in recent years a number of molecular genetic approaches have been developed in species with established molecular genetic systems, such as *Aspergillus nidulans*, to activate the expression of endogenous SM BGCs (Ahuja et al., 2012; Brakhage, 2013; Chiang et al., 2013, 2016; Grau et al., 2018, 2019; Henke et al., 2016; Keller, 2015; Kjærbølling et al., 2019a; Oakley et al., 2017; Yin et al., 2013; Yaegashi et al., 2014; Yeh et al., 2016; Yan et al., 2018, 2020). Efficient heterologous expression systems have been developed in some of these species to express BGCs from species that do not have established molecular genetic systems (Anyaogu and Mortensen, 2015; Chiang et al., 2013; Clevenger et al., 2017; Frandsen et al., 2018; Harvey et al., 2018; Kjærbølling et al., 2019a; Sakai et al., 2012; van Dijk and Wang, 2016). In spite of this progress, however, a major gap remains in our ability to exploit the greater fungal secondary metabolome efficiently. It is currently impossible to deduce the complete structure(s) of the compound(s) an SM BGC makes until it is synthesized; generally, we can only guess the class of compound the cluster produces. Additionally, the activities of compounds are discovered only after the compound's induction, purification, and extensive testing, all of which are very time consuming and labor intensive. There is a great need to be able to identify BGCs, among the millions present in fungi, that are likely to produce compounds with activities we desire.

Discoveries in our lab and others (Hutchinson et al., 2000; Hansen et al., 2011; Kennedy et al., 1999; Regueira et al., 2011; Yeh et al., 2016; Yan et al., 2018, 2020) have led to the development of a strategy that fulfills this need in many cases. This strategy, called SM resistance gene-guided genome mining, has enabled researchers to determine the targets of SMs produced by some fungal BGCs *in silico*, before their expression, isolation, and biological testing. This approach is based on the fact that some SM BGCs contain a gene that encodes a resistant form of the protein targeted by the compound produced by the BGC. The resistant allele is expressed along with the other genes of the BGC, and it confers resistance to the compound. This allows the compound-producing organism to survive while killing or inhibiting the growth of competing organisms. The *in silico* signature of such BGCs is that they contain an allele of an essential gene that has no SM biosynthetic function, and there is a second allele of that gene elsewhere in the genome. Other mechanisms of self-resistance to SMs exist, but, fortunately, resistance due to the presence of resistant alleles of essential genes in SM BGCs occurs frequently enough to potentially make this approach very useful.

Manually searching for this *in silico* signature is time consuming, and it is not practical at a large scale. At the time of this study, 1825 fungal genomes sequenced by the Joint Genome Institute were available on the MycoCosm web portal (mycocosm.jgi.doe.gov), and they contained 59 126 putative SM BGCs. The number of sequenced fungal genomes, fortunately, continues to increase rapidly, and a rapid, computer-guided method for resistance gene-guided genome mining is needed.

Currently, there are three published computer-guided approaches for resistance gene-guided genome mining in fungi (Kjærbølling et al., 2019b; Liu et al., 2021; Yılmaz et al., 2023). All are designed to be used with whole-genome sequencing data. In contrast, our goal has been to develop a process that enables users to query an annotated genomic database to find SM BGCs with resistance genes of our choice without downloading each individual genome. This allows the user to focus on genes that encode promising therapeutic targets. We have written a Python script that we have named rg3m, an abbreviation for **r**esistance **g**ene-**g**uided **g**enome **m**iner. This script allows us to identify instances in which a designated target gene is within a user-specified distance of an SM core biosynthetic gene, and (in the default setting) there are at least two copies of the target gene in the genome. rg3m has no additional assumptions. We have employed this approach with MycoCosm (https://mycocosm.jgi.doe.gov/mycocosm/home), a database of 1825 annotated fungal genomes, although, in principle, the approach would work with other databases of annotated genomes.

We have tested this approach by applying it to look for SM BGCs that target the proteasome β6 subunit or HMG-CoA reductase (HMGCR). The proteasome β6 subunit is the target of the proteasome inhibitor fellutamide B, and proteasome inhibitors have a growing number of therapeutic uses in cancer chemotherapy (Goldberg, 2012; Kisselev et al., 2012). HMG-CoA reductase is a key enzyme in sterol biosynthesis, and it is the target of cholesterol reducing therapeutics such as lovastatin (Alberts, 1990). Both are known to be produced by BGCs that contain resistant alleles of the target molecule (Hutchinson et al., 2000; Kennedy et al., 1999; Yeh et al., 2016) and if our approach is successful, we should find these known lovastatin-family and fellutamide family BGCs. We have found that our approach not only found known fellutamide and lovastatin BGCs, but previously undocumented examples as well. It also revealed BGC families that are closely related to documented fellutamide and lovastatin BGCs but with variations in the complement of SM genes that suggest that these BGCs could produce structural variants of fellutamides and lovastatin. Gratifyingly, we also found SM BGCs that are not closely related to lovastatin BGCs but putatively produce novel HMGCR inhibitors. In summary, our script is effective and easy to use, and it is not computationally heavy. It works in synergy with the accessible and user-friendly MycoCosm platform that has strong gene annotations and SM cluster prediction tools, allowing users to search for SM clusters harboring specific resistance genes without having to download entire genomes.

## Materials and Methods

### Script

Our script, rg3m, is available on github: https://github.com/apodgorny1/rg3m.

The script is in stock Python, such that it would be compatible with Python2 and Python3; it should run with the default library packages provided at installation. This obviates the need for any new libraries or their dependencies to be installed. Additionally, the code was written in an easy-to-understand way to allow modification to fit special use cases by users with little programming background. As there is variation in the gene database files available online, the most common headers were used for column inference allowing for flexibility of sources. This may be updated easily as required.

### Algorithm Complexity

As the core flow requires a loop over the entire set of the resistance genes, R, over each of the SM genes, S, the worst-case runtime complexity is O(RS). Memory complexity is the same, as both must be stored in memory simultaneously. If the homolog search is active, R is searched for each successful hit. In a case where every R is a hit, we add an O(R^2^) to the complexity. Homolog uniqueness is enforced by converting the final homolog list to a set.

### Pseudocode

Let: **R** be the set of resistance genes, **S** be the set of SM genes, *C* be the cutoff distance between gene centers, and *M* be the maximum gene length cutoff.

Start:

Generate **R** from resistance gene database, eliminating singletons if specified in parameters.

Generate **S** from SM gene database

For each r_gene in **R**:

For each s_gene in **S**:

Check if r_gene and s_gene are on the same organism.

Check if r_gene and s_gene are on the same scaffold.

Check if r_gene's and s_gene's centers are ⇐ to *C* nucleotides apart

Check that r_gene and s_gene do not overlap

Check that the resistance gene length is ⇐ to *M*

If all above criteria met:

Output to terminal and csv if specified.

Output resistance gene homologs if specified.

If homolog mode active:

Add all from **R** with the same organism to homolog table **H.**

End s_gene loop

End r_gene loop

Complete

### Genomic Data

Genomic data were obtained from the JGI MycoCosm database. Following the precedent of Reynolds et al. (2017), when a genome from MycoCosm had not been published and no special permission from the author(s) could be obtained, we referred to the genome only by taxonomic class and an assigned number. The data in the supplemental spreadsheets of this manuscript are based on pairwise BLASTP alignments using the default algorithm parameters for NCBI's online tool. Predicted protein amino acid lengths reported in this paper include the translation stop codon. At times, we used NCBI conserved domain searches and various NCBI BLAST search variations to gain additional understanding of some of the proteins under study. We also used the T-Coffee webserver (Di Tommaso et al., 2011) at http://tcoffee.crg.cat to create and analyze multiple sequence alignments (MSAs) of DNA and amino acid sequences to gain additional understanding of some of the DNA sequences and proteins under study.

## Results

### Algorithm and rg3m Computer Script

Since SM genes are often clustered around easily identifiable core backbone synthesis genes, we reasoned that we could circumvent potential errors inherent in approaches based on SM cluster prediction algorithms by simply determining if a target resistance gene is located within a specified distance of a core biosynthetic SM gene, and at least one additional allele of the target gene is present elsewhere in the genome. SM BGCs can vary greatly in size; for instance, the F9775/orsellinic acid BGC in *Aspergillus nidulans* is only 11 kb in length (Sanchez et al., 2010), whereas the aflatoxin cluster of *Aspergillus parasiticus* is ∼70 kb (Yu et al., 2004). In principle, the frequency of false positives (target resistance genes within the specified distance of a core biosynthetic gene even though there is no functional relationship between them) should increase as a function of the specified distance. Accordingly, we designed rg3m to allow the user to specify the maximum allowable distance between the center of the target resistance gene and the center of the closest core SM biosynthetic gene as defined by our input lists.

Our workflow involves four main steps. In step 1, we create a database of comma separated values (CSV) files containing the genomic coordinates of all the core SM biosynthetic genes (DMATs, PKSs, NRPSs, and terpene synthases/cyclases) in MycoCosm. A useful feature of MycoCosm is that one can choose to focus on a particular class or phylum, but for all cases described in this manuscript, we created files for all the sequenced fungi in MycoCosm. To generate these core biosynthetic gene CSV files, one can use a variety of search options such as a BLAST search, a keyword search, or a EuKaryotic Orthologous Groups (KOG terms) search as defined by MycoCosm. To generate our NRPS file we used KOG term 1178 to generate a CSV file of all of the “non-ribosomal peptide synthetase/alpha-aminoadipate reductase and related enzymes” on MycoCosm (27 807 genes). To generate our PKS file, we first did a trial run, generating three files using the search terms polyketide synthase, polyketide, and KOG1202 (“animal-type fatty acid synthase genes and related proteins”) and compared the results. The KOG1202 search gave the most complete file, yielding 27 249 genes. We have used it for all our analyses involving PKS genes in this paper and, henceforth, will refer to it as our PKS file. Using the search terms “terpene synthase,” “terpene cyclase,” and “tryptophan dimethylallyltransferase,” we generated files that contained 12 077 genes, 2436 genes and 3112 genes, respectively. While one could combine these files into a single large file containing all core biosynthetic genes, we have found it convenient to keep the various classes of core biosynthetic genes in separate files. These core biosynthetic gene files can be used for multiple searches and need only be updated from time to time as the number of sequenced fungal genomes increases.

In step 2, we create a CSV file containing the genomic coordinates for all the homologs of our target gene in a chosen fungal group (in our case all the fungi on MycoCosm). If, for example, we wish to find BGCs that produce an HMGCR inhibitor, we would create a file containing, in principle, all genes encoding HMGCR. Although one could use a variety of searches, we have found that TBLASTN searches generally give the most complete data sets.

**Fig. 1.**
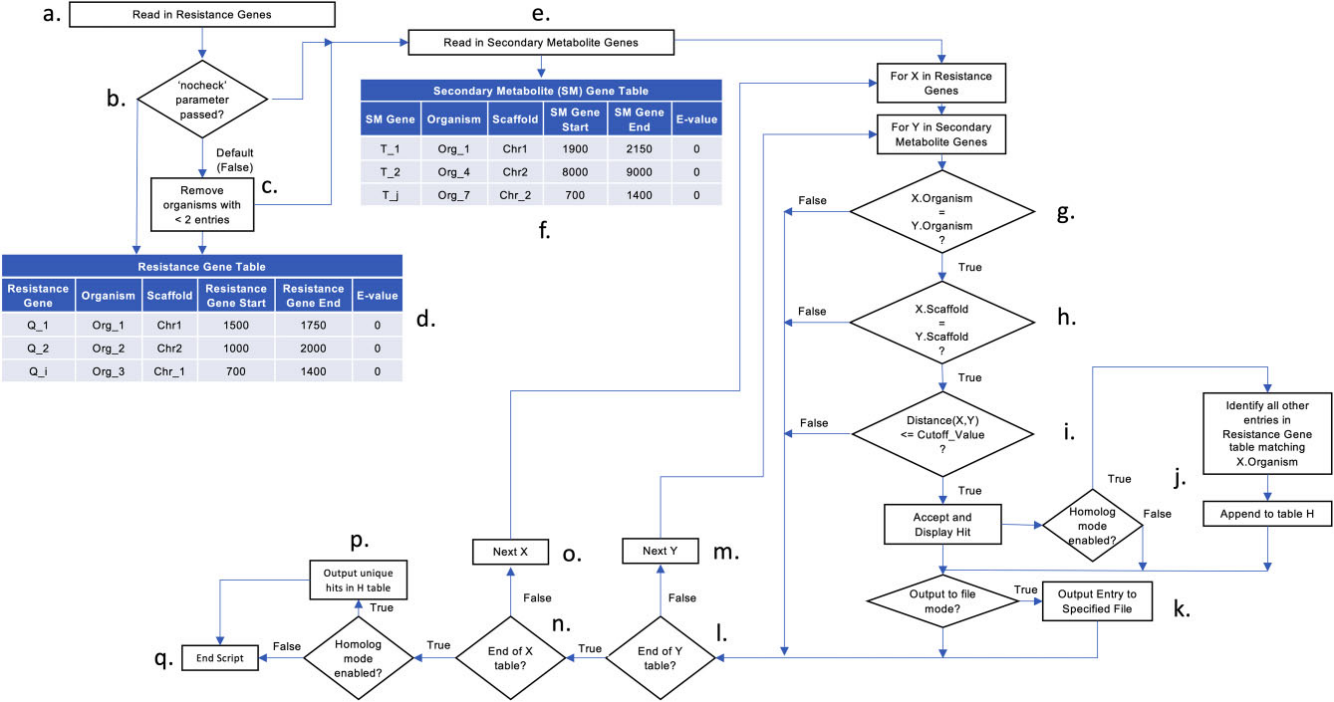
Flowchart of our resistance gene-guided genome mining script, rg3m. The script initially reads a CSV file of all query genes (housekeeping and putative resistance genes) (**a**). If desired, the “nocheck” parameter (**b**) can be implemented to waive the requirement for each genome to contain at least two query genes (a housekeeping and putative resistance gene). This would preclude resistance gene-guided genome mining, but it might have other uses. One could, for example, find all the transcription factors within a user-specified distance of a secondary metabolism core biosynthetic gene. Normally, genomes in which there are fewer than two query genes are removed (**c**), resulting in a virtual table containing putative resistance genes along with the assembled genomes in which they are found (organism), as well as their scaffold numbers and genomic coordinates (**d**). Next, the script reads in the secondary metabolite gene data (**e**), creating a virtual secondary metabolite gene table that contains the genomic coordinates of secondary metabolism core biosynthetic genes (**f)**. For each putative resistance gene, the script interrogates the secondary metabolite gene, determining if the resistance gene is in the same genome (organism) (**g**). If it is not in the same genome, the script moves to the next gene in the secondary metabolite gene table. If it is in the same genome, the script determines if the resistance gene is in the same scaffold (**h**), and if it is in the same scaffold, it determines if it is within a user-specified distance of the target SM gene **(i**). If it is within that distance, the script accepts it as a hit. If the homolog mode is active, any resistance genes with a matching organism are added to a Homolog table (**j**). If the output to file mode is activated, the hit is outputted to a user-specified file (**k**). If the query gene is not on the same scaffold as the target gene, or it is not within a user-specified distance of the target gene, the script moves to the next target gene in the Secondary Metabolite table (**l, m**). This is repeated for all genes in the secondary metabolite gene table until the end of the table is reached. The process is repeated for the next resistance gene (**n, o**) until all resistance genes have been exhausted. If the homolog mode is active, any homologs are output to the specified file (**p**). The script is then ended (**q**).

In step 3, each core biosynthetic gene file and the target gene file are used as input data by rg3m (Fig. 1). rg3m queries the two files, looking for instances in which two or more homologs of the target resistance gene are present in the same genome and one of the homologs is within a user-specified distance of a core SM biosynthetic gene. (Note, in this instance the distance is defined as the distance between the center of the target gene and the center of the core biosynthetic gene as designated by their genomic location in the input files. In all other cases in this manuscript, when we refer to distances between genes, we refer to the distances between the ends of genes as defined by the beginnings and ends of genes as annotated in MycoCosm.) The rg3m script creates a list of ‘hits’ which can be output into a CSV file at the user's command. This list contains the genome designation as specified in the MycoCosm database (which can include the species, strain, and annotation version), the *E*-value and percent identity of the predicted protein product of the putative resistance gene relative to the homolog used as the query for the TBLASTN search, the genomic coordinates of the resistance gene and the core backbone SM biosynthesis gene, and the distance between the resistance gene and core backbone synthesis gene. In step 4, the researcher evaluates these hits manually, aided by various tools such as the genome browser and SMURF SM cluster predictions available on MycoCosm.

To determine the efficacy of our approach, we tested it by looking for BGCs that putatively produce inhibitors of the β6 subunit of the proteasome or of HMGCR. The proteasome is a critical cellular complex that is required for proteolysis of proteins targeted for destruction by ubiquitination. Proteasome inhibitors are increasingly valuable cancer chemotherapy agents (Goldberg, 2012; Kisselev et al., 2012), and the proteasome inhibitor fellutamide B is a potent inhibitor of the tuberculosis-causing bacterium *Mycobacterium tuberculosis* (Lin et al., 2010). Fellutamide B inhibits the proteasome by targeting the β6 proteasome subunit, and the *A. nidulans* fellutamide B BGC contains a β6 homolog (*inpE*) that has been demonstrated experimentally to confer resistance to fellutamide B (Yeh et al., 2016). HMG-CoA reductase is a critical gene in sterol biosynthesis in both fungi and humans. It is inhibited by lovastatin, and the *Aspergillus terreus* lovastatin BGC contains an HMGCR allele (*lvrA*) that confers resistance (Hutchinson et al., 2000). Fellutamide B and lovastatin clusters have been reported in several fungi in the MycoCosm database. We reasoned that if our approach works, known fellutamide B and lovastatin clusters should be included among our hits. Additionally, previously undiscovered fellutamide-like or lovastatin-like clusters might be found. Perhaps most interestingly, we anticipated our approach might find BGCs that putatively encode novel classes of inhibitors of the proteasome β6 subunit or HMGCR.

We also reasoned that analyzing the distinct cluster families whose members produce similar SMs could help us improve our algorithm and understand this unique class of SM BGCs that have resistance genes. For instance, our analysis of these clusters might teach us more about the sizes and gene arrangements of SM BGCs with resistance genes, which could aid us in setting generalized input parameters or optimizing our algorithm. Studying these clusters might also be informative to the evolutionary inheritance of SM BGCs among fungi, that is, horizontal gene transfer.

### Proteasome β6 Subunit BGCs

We used the “housekeeping” β6 subunit proteasome gene AN5784 from *A. nidulans* (FungiDB.org) (Arnaud et al., 2012; Yeh et al., 2016) and an *E*-value cutoff of 1E-40 to conduct a TBLASTN search of fungal genomes in MycoCosm. This generated a list of 1557 β6 subunit proteasome genes, and their coordinates. Inputting our NRPS and β6 subunit proteasome files into the rg3m script with a distance cutoff value of 70 kb generated a list of 81 hits in 42 genomes, with two hits representing the *A. nidulans* fellutamide B BGC (Supplementary Spreadsheet 1, NRPS Hits at 70 kb tab). Note that because fellutamide clusters contain two NRPS genes, each complete fellutamide BGC generates two hits.

**Fig. 2.**
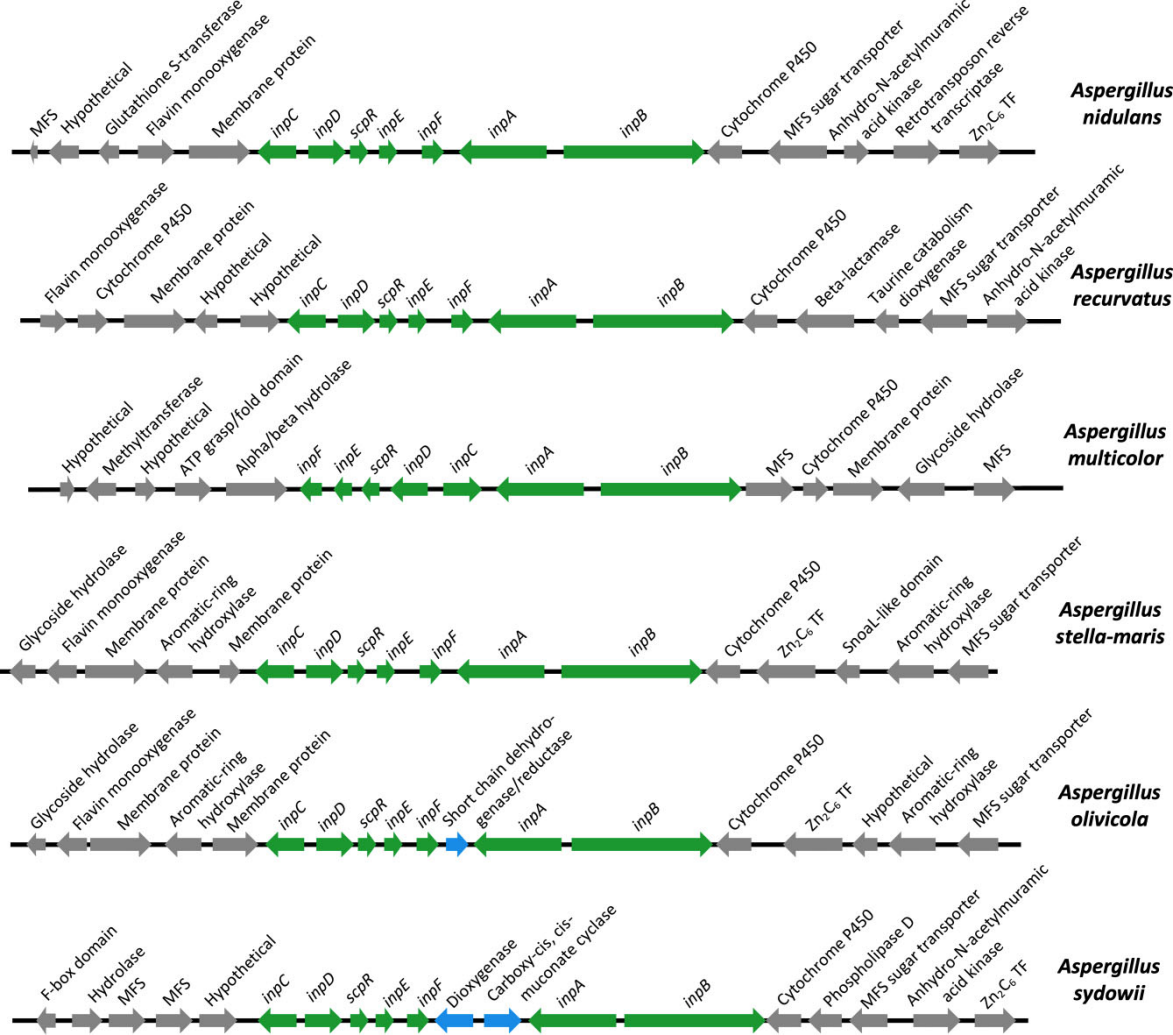
Representative fellutamide family BGCs in the genus *Aspergillus.* The species is shown at the right of each line. Arrows show the direction of transcription of each gene. They are not drawn to precise scale. The green arrows in the *Aspergillus nidulans* cluster represent genes that are functional components of the BGC (Yeh et al., 2016). The proposed functions of the proteins they encode are: *inpA =* NRPS, *inpB* = NRPS, *inpC =* fatty-acyl-AMP ligase, *inpD* = transporter, *inpE* = proteasome β6 subunit homolog (resistance gene), *inpF* = esterase or lipase involved in NRPS product release/transfer, *scpR* = transcription factor. The homologs of these genes in the other BGCs have been labeled with the *A. nidulans* gene symbols and percentage identities are given in Supplementary Spreadsheet S1, Fellutamide Cluster BLASTPs tab. The BGCs are highly conserved although the gene order is not absolutely consistent. Blue arrows represent genes that are within the BGC but do not have homologs in the *A. nidulans* BGC. They have predicted products with potential functions in secondary metabolism and, therefore, they could participate in the production of a modified fellutamide. Five flanking genes on each side of the BGCs are shown. The flanking regions of *A. stella-maris* and *A. olivicola* are highly conserved but the flanks of the other clusters are less conserved. Many of these genes also have potential functions in secondary metabolism and could, in principle, alter the final product of the BGC.

Next, using MycoCosm's genome browser, we reviewed the hits by looking at the annotations and ontologies of the genes in the regions around the putative NRPS and proteasome subunit genes. The fellutamide B cluster in *A. nidulans* (Fig. 2) contains two NRPS genes, a fatty-acyl-AMP ligase gene, a transporter gene, a TF gene, a proteasome β6 subunit homolog, and an esterase or lipase gene, the product of which is predicted to be involved in product release/transfer (Yeh et al., 2013). For one hit, in *Amylomyces rouxii* NRRL 5866 v1.0 (Wang et al., 2023), due to the presence of a large number of genes with unknown functions in the region, it was not obvious to us that the genes around the proteasome β6 formed an SM BGC. There were three hits in the *Aspergillus multicolor* v1.0 genome. Two hits were NRPS genes that formed part of a fellutamide cluster, but the third hit was an NRPS gene located almost 40 kb away from this fellutamide cluster. The clusters in the remaining genomes, however, were clearly complete or partial fellutamide clusters, based on the genes they contained and pairwise BLASTP comparisons that revealed the high degree of homology of the predicted products of those genes (low *E*-values, high percentage similarity, and high percentage identity) to the proteins encoded by homologous *A. nidulans* fellutamide cluster genes (Supplemental Spreadsheet 1, Fellutamide Cluster BLASTPs tab).

Twenty-nine *Aspergillus* genomes in addition to the *A. nidulans* genome contained fellutamide BGCs with homologs of all the genes present in the *A. nidulans* cluster (Supplementary Spreadsheet 1, Fellutamide Cluster BLASTPs tab and examples in Fig. 2). *Aspergillus navahoensis* v1.0 contained an incomplete fellutamide cluster lacking homologs of the of the *inpA* NRPS gene and the *inpF* product release/transfer gene. BLAST searches did not reveal strong homologs of these genes elsewhere in the genome, so the absence of these genes did not appear to be a genome assembly error. The NRPS gene that was present was a homolog of *inpB* but was much shorter, with a predicted product of 688 amino acids versus 2327 amino acids for *inpB*. The smaller gene did not appear to be an annotation error. Rather, the 5’ end of the gene appeared to be deleted. This cluster could, thus, be an evolutionary relict rendered dysfunctional by a deletion that removed a portion of the cluster.

The hits in *Aspergillus fructiculosus* v1.0 and *Aspergillus falconensis* CBS 271.91 v1.0 genomes were in clusters very similar to each other (Supplementary Spreadsheet S1, Fellutamide Cluster BLASTPs tab and Supplementary Fig. S1). The cluster was near the end of a scaffold in each genome with the scaffolds extended in opposite directions from a syntenic region. Within the syntenic region there were genes putatively encoding an AMP-dependent synthetase/ligase NRPS, a proteasome subunit, which appeared to be fused to a TF gene, a transporter gene, a TF gene, and a fatty-acyl-AMP ligase gene, each with strong homology to genes in the *A. nidulans* fellutamide cluster (Supplementary Spreadsheet S1, Fellutamide Cluster BLASTPs tab). The genes were in the same order in the two clusters. We looked for *inpA* and *inpB* homologs in regions upstream and downstream of the BGCs, and they were absent. The syntenic region did contain genes encoding a monooxygenase and cytochrome P450 which often function in biosynthesis of SMs. These two clusters are clearly related to fellutamide BGCs but could not make fellutamides (assuming that the absence of other fellutamide BGC genes is not due to incorrect genome assembly), although they might make other products.

*Trichocoma paradoxa* CBS 103.73 v1.0, *Bisporella* sp. PMI857 v1.0, and *Spathularia flavida* all had complete clusters. Note that the TF in *S. flavida* is not annotated in MycoCosm and that fellutamide clusters have been previously identified in *Bisporella* sp. PMI857 v1.0 and *S. flavida* (Lan et al., 2020). A fellutamide cluster is present in the lichen fungus *Lobaria pulmonaria* (Supplementary Spreadsheet S1, Fellutamide Cluster BLASTPs tab and Supplementary Fig. S2) as previously noted (Lan et al., 2020), and it merits additional comment. The cluster is present in the reference strain isolated in Scotland and in extracted genomes from Africa, Spain, Scotland, and Switzerland (i.e., genomes deduced from the sequenced lichen, including symbionts, rather than the purified fungus). The cluster lacks the NRPS product release/transfer gene *inpF* found in *A. nidulans*. However, in *A. nidulans, inpF* is not strictly required for fellutamide B production, but production is greatly reduced in its absence (Yeh et al., 2016). Given that lichens grow very slowly, it is possible that slow production of metabolites might be advantageous to match the growth rate and maintain appropriate levels in thalli. We noticed two additional oddities about the *L. pulmonaria* data. One is that the annotated versions of the proteasome subunit in the cluster appear shorter than in other organisms. However, there are sequences upstream of the putative start codon that are strongly homologous to the N-terminal regions of the predicted protein in other genomes in this study. This raises the possibility of a sequencing and/or annotation error, but a second oddity is that the sequences of the region encoding the subunit are extraordinarily conserved at the nucleotide level even though the sequenced samples were collected from geographically distant locations. For instance, in an MSA of a region spanning from 750 nucleotides upstream of the start codon of the proteasome subunit gene to 750 nucleotides downstream of the start codon of the proteasome subunit gene from the five *L. pulmonaria* genomes, we found only five instances where nucleotides differed. Note, however, that there is less conservation in the regions flanking the BGC (Supplementary Fig. S2).

Finally, it is worth noting that some of the fellutamide gene clusters have adjacent genes that potentially have roles in secondary metabolism (examples are shown in Fig. 2), and a small number have additional potential secondary metabolism genes within the BGCs. It is possible that the products of these genes may modify the products of the BGCs, creating fellutamide variants.

Having confirmed the effectiveness of our approach in finding fellutamide clusters, we searched for other SM BGC classes looking for clusters that produce new classes of β6 proteasome subunit inhibitors. Using the rg3m script to query our PKS file with a distance cutoff of 70 kb revealed only seven hits (Supplementary Spreadsheet 1, PKS Hits at 70 kb tab). In two of these, *Bisporella* sp. PMI857 v1.0 and *Aspergillus recurvatus* v1.0, the PKS was within 70 kb of a β6 proteasome subunit in a fellutamide-family cluster we had found in our NRPS search. There were approximately 15 genes between the PKS and the core fellutamide BGC in each case, and the putative functions of several of these genes suggested a role in secondary metabolism. The distributions of SM and non-SM genes suggested, however, that in each case the PKS gene is likely to be a part of a separate SM BGC that is near to the fellutamide-family BGC rather than a component of a giant fellutamide-family BGC.

In four hits (*A. fructiculosus* v1.0*, A. falconensis* CBS 271.91 v1.0, Leotiomycetes Genome 1 and Agaricomycetes Genome 3), the PKS genes appeared to be in BGCs, but there were several genes with no apparent role in secondary metabolism between the proteasome subunit gene and the PKS. In these instances, the reason for the proximity of the proteasome subunit to the BGC is unknown.

The remaining hit was in an unpublished Eurotiomycetes Genome (Eurotiomycetes Genome 1, Supplementary Spreadsheet 1, PKS Hits at 70 kb tab) in which the proteasome subunit gene is located 18 598 bp away from the PKS gene. The two genes are flanked by several genes with putative functions in secondary metabolism, suggesting that they are in a BGC and raising the possibility that the BGC makes a novel polyketide proteasome inhibitor. The validity of the BGC is called into question, however, by the fact that there are a number of hypothetical genes with no predicted function in the region, including some between the PKS gene and the proteasome subunit gene. If the large number of hypothetical genes is simply due to poor annotation and gene calling and the BGC is valid, this could be an exciting BGC to investigate further.

To determine if terpene synthases might be backbone genes of other BGCs that produce proteasome β6 inhibitors, we ran the rg3m script, again with a distance cutoff of 70 kb, and this returned only three hits (in *Postia placenta* MAD 698-R v1.0 (Martinez et al., 2009), *Suillus occidentalis* FC124 v1.0 (Lofgren et al., 2021), and *Suillus lakei* FC43 v1.0 (Lofgren et al., 2021) (Supplementary Spreadsheet 1, Terpene Synthase Hits at 70 kb tab). In each case, there was no obvious SM BGC around the terpene synthase, and they were, thus, not particularly good candidates for producing proteasome β6 inhibitors. Finally, querying our terpene cyclase and DMATS databases for proteasome β6 inhibitors using a 70 kb cut off yielded no hits.

### HMG-CoA Reductase Inhibitors

#### Lovastatin family BGCs

To test our approach further, we looked for BGCs containing HMGCR genes, hoping to find known and, potentially, undiscovered lovastatin-like clusters, and other clusters that potentially produce new HMGCR inhibitors. To construct our HMGCR homolog file, we used the *A. terreus* NIH 2624 (Arnaud et al., 2012) HMGCR housekeeping gene (ATET_09520) to conduct a TBLASTN search of the MycoCosm database using an *E*-value cut off of 1E-40. This search generated a CSV file containing 2326 HMGCR homologs. Interrogating the HMGCR homolog file and our PKS file with the rg3m script, using a cutoff value of 70 kb, generated 85 hits including two hits representing the *A. terreus* NIH 2624 lovastatin cluster (the lovastatin BGC contains two PKS genes) (Supplementary Spreadsheet 2, PKS Hits at 70 kb tab).

**Fig. 3.**
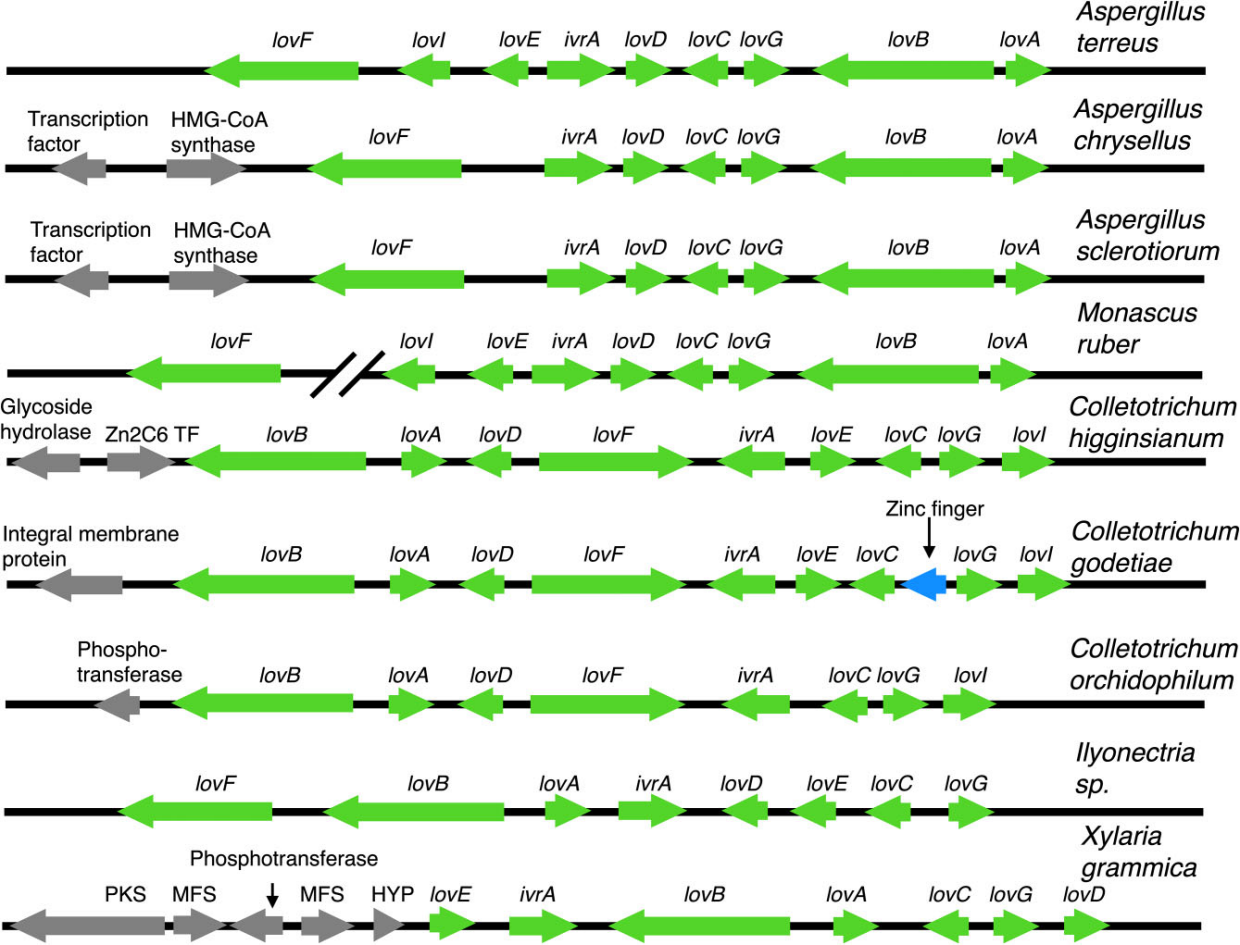
The order and orientation of genes within representative lovastatin family BGCs. The species is shown at the right above each line. Arrows represent genes and their directions of transcription. Green arrows are putative members of lovastatin BGCs, and gray arrows are genes adjacent to the BGCs that are of potential interest with respect to secondary metabolism. Gene sizes and intergenic distances are not to scale. The top line shows the lovastatin BGC of *Aspergillus terreus*. Gene symbols are given for each gene. The predicted biochemical functions for the products of the genes are as follows: *lovA* = P450 monooxygenase; *lovB* = polyketide synthase; *lovC* = dehydrogenase; *lovD* = transesterase; *lovE* = transcription factor; *lovF* = polyketide synthase; *lovG* = oxyreductase; *lovI* = efflux pump; *IvrA* = HMG-CoA reductase gene (resistance allele). For the other species, homologs of the *A. terreus* genes are labeled with the *A. terreus* gene symbols. Percentage identities are given in Supplementary Spreadsheet S2, Lovastatin BLASTPs tab. The gene order and orientation of the *Aspergillus chrysellus* and *Aspergillus sclerotiorum* BGCs are identical to each other, and they are the same as for *A. terreus* except that they do not have homologs of *lovE* or *lovI*. They each have a transcription factor very near the BGC but whether the transcription factor functions in expressing the BGC is unknown. There are many MFS genes in the genome that could encode MFSs that carry out the function of the missing *lovI. Monascus ruber* has a very similar BGC to the *Aspergillus* species but it was on the edge of a scaffold and the *lovF* homolog was on a different scaffold. The three *Colletotrichum* BGCs have similar gene orders to each other, but they are different from the *Aspergillus* species gene orders, and they have different genes flanking the BGC. The gene order of the *Ilyonectria sp.* Biosynthetic gene clusters is different from the others but there are good homologs of all of the *A. terreus* genes except *lovI*, the efflux pump. *Xylaria grammica* does not have good homologs of *lovF* and *lovI*, but it has two MFS genes and a PKS nearby that could, in principle, be part of the BGC, functioning in place of *lovF* and *lovI.* Notes: There is a putative Zinc finger encoding gene (blue arrow) between the *lovC* and *lovG* homologs in *Colletotrichum godetiae*, although it was not present in the other BGCs. HYP in *Xylaria grammica* stands for hypothetical gene.

Using MycoCosm's genome browser, we reviewed the hits by examining the gene annotations and ontologies of the genes in the regions around the putative PKS and HMGCR genes. In addition to the two *A. terreus* NIH 2624 hits, 18 hits in 10 genomes were in BGCs that resembled the *A. terreus* lovastatin cluster (Supplementary Spreadsheet 2, PKS Hits at 70 kb tab and Lovastatin Cluster BLASTPs tab; examples are shown in Fig. 3). Because the lovastatin cluster contains two PKS genes, our script generated two hits for most lovastatin family clusters. The exceptions were: *Colletotrichum godetiae* CBS 193.32 v1.0, which generated three hits because the *lovF* homolog was incorrectly annotated and split into two genes, *Xylaria* sp. FL1777 v1.0 (Franco et al., 2022), which generated only one hit because it does not have the *lovF* PKS (BLASTP searches did not reveal a strong *lovF* homolog anywhere in the genome), and *Monascus ruber* NRRL 1597 v1.0, which generated only one hit because the cluster was located at the edge of a scaffold, and this resulted in the *lovF* homolog being located on a different scaffold (we subsequently found the *lovF* homolog through a BLASTP search). *Aspergillus homomorphus* CBS 101889 v1.0 (Vesth et al., 2018) generated only one hit because it has a partial HMGCR cluster, containing only three annotated genes, one of which is a homolog of *lovF* (Supplementary Spreadsheet 2, Lovastatin Cluster BLASTPs tab). A TBLASTN search of the genome did not reveal strong homologs of the missing genes, and they are, thus, likely to be genuinely absent rather than separated from the remainder of the cluster by incorrect or incomplete genome assembly.

Expression studies, deletion studies, and comparisons of the lovastatin/monocolin K gene clusters from *A. terreus* and *Monascus pilosus* have revealed that the lovastatin cluster in *A. terreus* contains the aforementioned two PKS genes (*lovB* and *lovF*), a major facilitator superfamily (MFS) gene (*lovI*), an HMGCR gene (*lvrA*), an acyl transferase gene (*lovD)*, an enoyl reductase gene (*lovC*), a thioesterase gene (*lovG*), a P450 monoxygenase gene (*lovA*), and a TF gene (*lovE*) (Hutchinson et al., 2000; Kennedy et al., 1999; Sorensen et al., 2001; Sorensen and Vederas, 2003; Chen et al., 2008). We performed pairwise BLASTP comparisons of the genes surrounding each of our hits against their potential *A. terreus* NIH 2624 cluster homologs (Supplementary Spreadsheet 2, Lovastatin Cluster BLASTPs tab). The BLASTP comparisons showed a high degree of homology (low *E*-values, high percentage similarity, and high percentage identity) confirming that these BGCs are closely related to the *A. terreus* lovastatin BGC, further validating our approach (Supplementary Spreadsheet 2, Lovastatin Cluster BLASTPs tab). There were differences in the compositions and gene order of the BGCs, however (Fig. 3), indicating that the BGCs have changed over time.

*Colletotrichum higginsianum* IMI 349063 (Zampounis et al., 2016), *C. godetiae* CBS 193.32 v1.0 and *M. ruber* NRRL 1597 v1.0 had strong homologs of all of the genes in the *A. terreus* NIH 2624 lovastatin BGC, although in *C. godetiae* CBS 193.32 v1.0 the *lovF* PKS gene was misannotated as two genes. As mentioned, the *A. homomorphus* CBS 101889 v1.0 genome contained a partial BGC with only three annotated genes, homologs of *lovD* (acyl transferase)*, lvrA* (HMGCR), and *lovF* (PKS) (and potentially the transcription factor *lovE*, although the TF was not annotated as a gene in MycoCosm). Since this cluster does not have the *lovB* PKS gene, it cannot make lovastatin, and it may be a remnant of a once functional lovastatin cluster. The BGCs in *Ilyonectria* sp. MPI-CAGE-AT-0134 v1.0 (Mesny et al., 2021), *Aspergillus chrysellus* CBS 472.65 v1.0, *Aspergillus sclerotiorum* CBS 549.65 v1.0, *A. homomorphus* CBS 101889 v1.0, *Xylaria grammica* CBS 120713 v1.0 (Franco et al., 2022) and *Xylaria* sp. FL1777 v1.0 genomes lacked homologs of the MFS gene ATET_09967 (*lovI).* Major facilitator superfamily proteins are membrane proteins that move small solutes across membranes. In lovastatin BGCs, the MFS is likely to export the compound produced by the BGC (i.e., lovastatin or a closely related compound). There are numerous MFS genes in fungal genomes, and, in instances in which there is no MFS gene in the BGC, this function may be carried out by an MFS separate from the BGC.

The *lovC* enoyl reductase gene in *Colletotrichum orchidophilum* IMI 309357 (Baroncelli et al., 2018) was not found in the same stretch of DNA as the rest of the lovastatin genes, but we found a strong homolog elsewhere in the genome through a BLASTP search. We found two strong *lovG* homologs in *C. orchidophilum* IMI 309357. One homolog was located in the same area as the majority of the genes in the lovastatin cluster, and, as annotated, it encodes a predicted protein of 824 amino acids in comparison to the 243–264 amino acids predicted in other species. The abnormal length appears to be due to an incorrect annotation that fuses the *lovG* homolog with the homolog of the *lovI* MFS gene. The second *lovG* homolog in *C. orchidophilum* IMI 309357 was found with a BLASTP search. It was located next to the *lovC* enoyl reductase gene, and the pair were separated in the genome from the remainder of the BGC. This suggests that a portion of the *C. orchidophilum* IMI 309357 lovastatin BGC has been duplicated and translocated.

Pairwise BLASTP comparisons of the LovF PKS with its homologs revealed strong homology in most cases (Supplementary Spreadsheet 2, Lovastatin Cluster BLASTPs tab). Exceptions were *Xylaria* sp. FL1777 v1.0 that does not have a homolog, *X. grammica* CBS 120713 v1.0 in which the closest LovF homolog was only 32% identical to LovF, and *Ilyonectria* sp. MPI-CAGE-AT-0134 v1.0, in which the closest homolog was only 29% identical. LovF produces a side chain that is added to the core lovastatin scaffold. We speculate that the variability in the LovF homologs may result in different hydrocarbon chains being produced, particularly for the *Ilyonectria* sp. MPI-CAGE-AT-0134 v1.0 homolog, which has relatively low identity with LovF. It is also likely that the absence of a *lovF* homolog in *Xylaria* sp. FL1777 v1.0 results in production of monacolin J, a lovastatin-like compound that simply lacks this side chain but still retains HMGCR inhibitory activity (Endo et al., 1985). Finally, some of these lovastatin family BGCs contain, or are adjacent to, additional genes with likely roles in secondary metabolism. These genes may encode proteins that modify the lovastatin-like compounds to produce additional variants of lovastatin. These genes did not appear, however, to be conserved among the BGCs or among subsets of the BGCs.

Examination of BGCs and BLASTP comparisons provide evidence for the divergence of regulatory mechanisms (Supplementary Spreadsheet 2, Lovastatin Cluster BLASTPs tab). The BGCs in *C. orchidophilum* IMI 309357, *A. chrysellus* CBS 472.65 v1.0, and *A. sclerotiorum* CBS 549.65 v1.0 lacked a homolog of the TF (*lovE*) gene and, therefore, must be regulated by TFs encoded by genes outside the cluster.

### Non-lovastatin Family BGCs With PKS Backbones

BLASTP comparisons and thorough analyses of the regions surrounding the remaining 65 PKS hits confirmed that they are not part of lovastatin-family BGCs. We note, however, that in many, but not all, instances the HMGCR and PKS genes are clearly in SM gene clusters. Given the relatively low rate of false positives we see with our proteasome β6 subunit search (discussed above), these data raise the exciting possibility that these BGCs may produce new classes of HMGCR inhibitors with polyketide backbones.

**Fig. 4.**
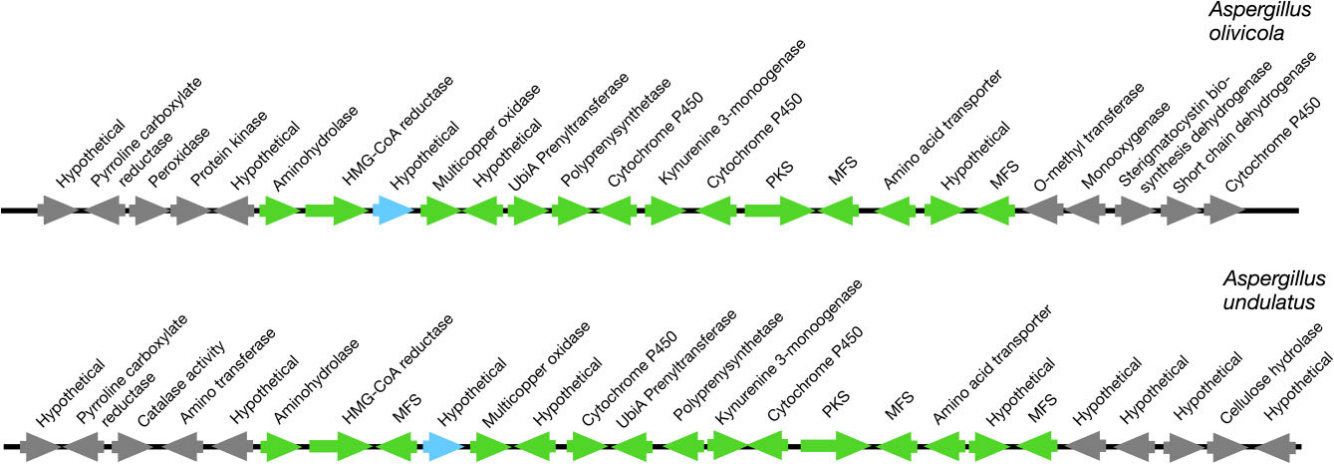
Two closely related non-lovastatin BGCs predicted to produce novel HMG-CoA reductase inhibitors. Arrows show genes (not to scale) and direction of transcription. Green arrows are putative members of the BGCs, and gray arrows are genes in flanking regions. The blue arrows are both hypothetical proteins, but they don't show strong identities with each other.

While many of these BGCs are unique, without close relatives in MycoCosm (see examples in Supplementary Fig. S3), others have sibling clusters containing similar complements of homologous SM genes, forming, in aggregate, families of clusters. *Aspergillus olivicola* v1.0 and *Aspergillus undulatus* CBS 261.88 v1.0, for example, have very similar clusters containing genes putatively encoding an amidohydrolase, a multicopper oxidase, a polyprenyl synthetase, a prenyl transferase, an NAD(P)/FAD-dependent oxidoreductase, two cytochrome P450s, two MFS proteins, two conserved hypothetical genes, and an amino acid transporter in addition to the PKS and HMGCR genes (Supplementary Spreadsheet 2, *A. olivicola* Family tab and Fig. 4.) Pairwise BLASTP comparisons of homologs from these two clusters showed strong homology with extremely low *E* values and high percentages of identity and similarity, confirming that they are closely related. Genes on the right flank of the two BGCs have little homology. A pyrroline carboxylate reductase gene is present in both left flanks, but other genes differ. Overall the data are consistent with the BGC being inherited as a unit as would be expected for an SM BGC.

Similarly, *Aspergillus transmontanensis* CBS 130015 v1.0 (Kjærbølling et al., 2020) and *Aspergillus parasiticus* CBS 117618 v1.0 (Kjærbølling et al., 2020) have clusters containing, in addition to the PKS and HMGCR genes, genes putatively encoding a PKS dehydratase domain protein, an MFS protein, an oxidoreductase with a transmembrane domain and a PKS enoyl reductase domain protein (Supplemental Spreadsheet 2, *A. transmontanensis* Family tab). Pairwise BLASTP comparisons of homologs from these clusters showed very strong homology *Aspergillus minisclerotigenes* CBS 117635 v1.0 (Kjærbølling et al., 2020) has a truncated version of this cluster, only containing genes for the PKS, HMGCR, and PKS dehydratase domain proteins. As noted for previous examples, this apparent truncation could be a genome assembly artifact.

Finally, *Aspergillus uvarum* CBS 121591 v1.0 (Vesth et al., 2018), *Aspergillus violaceofuscus* CBS 115571 v1.0 (Vesth et al., 2018), and *Aspergillus japonicus* CBS 114.51 v1.0 (Vesth et al., 2018) contained putative clusters containing genes encoding NADH-dehydrogenase, FAD oxidase, a PKS, two Zn2Cys6 TFs, an actin depolymerizing factor (ADF, also known as cofilin), an acyl transferase, a cytochrome P450, and HMGCR (Supplementary Fig. S4). BLASTP comparisons of these proteins with their *A. japonicus* CBS 114.51 v1.0 cluster homologs revealed extremely strong homology (Supplementary Spreadsheet 2, *A. japonicus* Family tab and Supplementary Fig. S4). *Aspergillus aculeatus* ATCC16872 v1.1 (de Vries et al., 2017) has a similar cluster, but it lacks the FAD oxidase and one of the two Zn2Cys6 TFs. With the exception of ADF, the genes are typical of those found in SM BGCs, so these appear to be *bona fide* SM BGCs. There are, however, sterol biosynthesis genes in addition to HMGCR nearby in the genomes in each of these cases. This raises the slight possibility that these clusters are located in genomic regions that are simply enriched for genes involved in sterol biosynthesis, and that the HMGCR gene is associated with the cluster by chance. The regions flanking the putative BGC are distinct in the four genomes and this helps define the boundaries of the BGC. There are, however, some homologs in the flanks that are shared among flanks of two or more species. This is consistent with the BGC being inherited vertically with genes in the flanking regions changing over time.

The conserved presence of an ADF gene, which is non-SM-biosynthetic, in the midst of these clusters raises additional questions. Actin depolymerizing factors are conserved in eukaryotes and ADF function (actin depolymerization) is essential. Actin depolymerizing factor inhibitors would certainly be toxic to non-resistant competitors. Phallotoxins produced by basidiomycetes of the genus *Amanita*, for example, stabilize F-actin, preventing disassembly (Wieland, 1983). We have looked for other ADF alleles in the *A. uvarum* CBS 121591 v1.0, *A. violaceofuscus* CBS 115571 v1.0, *A. aculeatus* ATCC16872 v1.1, and *A. japonicus* CBS 114.51 v1.0 genomes, and each genome contains two ADF homologs in addition to the one in the BGC. These data raise the possibility that these BGCs produce an ADF inhibitor rather than an HMGCR inhibitor and that the ADF gene in the cluster confers resistance. Without additional data, we cannot, of course, rule out the possibility that the cluster produces both ADF and HMGCR inhibitors or a single compound that inhibits both.

### Putative HMG-CoA Reductase Inhibitor BGCs With NRPS Backbones

After confirming our approach through finding lovastatin family clusters and new families of PKS clusters that putatively produce new HMGCR inhibitors, we expanded our search to other classes of core biosynthetic genes. Querying our NRPS database using a distance of 70 kb returned 95 hits (Supplementary Spreadsheet 2, NRPS Hits at 70 kb tab). Inspection of the regions around the NRPS and HMGCR genes revealed that, while some of these hits do not appear to be in BGCs, or there are multiple non-SM genes (genes with no feasible role in secondary metabolite production) between the NRPS and HMGCR genes, many of them appear to be in valid BGCs with the HMGCR gene contiguous with the remaining genes of the BGC. They are, thus, excellent candidates for BGCs that produce novel classes of HMGCR inhibitors.

There was a notable family of 19 BGCs in the basidiomycete genera *Pisolithus, Boletus, Rickenella, Suillus*, and two different genera from unpublished Agaricomycetes (Supplementary Spreadsheet 2, Pisolithus Family tab. Several of the species in this group are dikaryons. This may explain why there are two similar, but not identical, clusters on scaffolds 5 and 14 in *Pisolithus croceorrhizus* subspA 74A v1.0 (Plett et al., 2023) and two on scaffolds 58 and 65 in *Pisolithus thermaeus* 11 v1.0 (Plett et al., 2023). In addition to the NRPS gene, all of these BGCs contain genes that putatively encode an alcohol dehydrogenase, an oxidoreductase with an NAD(P) binding domain, HMGCR, and at least one AMP synthetase/ligase (Supplementary Spreadsheet 2, Pisolithus family tab). Many also contain a putative multicopper oxidase gene, a fatty acid hydroxylase gene, and a second AMP synthetase/ligase. BLASTP comparisons of the predicted products of these genes, revealed that the homologs in these BGCs are highly conserved. Interestingly, all but two of the BGCs contain a gene encoding a homolog of the exocyst component Sec5, a protein that is unlikely to function in SM biosynthesis. This raises the possibility that these BGCs could make a Sec5 inhibitor with the sec5 allele in the BGC conferring resistance. In aggregate, these results suggest that these clusters are a family of related BGCs. They may produce a new class of HMGCR inhibitors that have a common backbone structure and related, but probably not identical, final products. Interestingly, members of the genera *Boletus* and *Suillus* are edible, raising the possibility that consuming them might have some cholesterol-reducing effect.

### Putative HMG-CoA Reductase BGCs With Terpene synthase, Terpene cyclase, and DMATS Backbone Genes

**Fig. 5.**
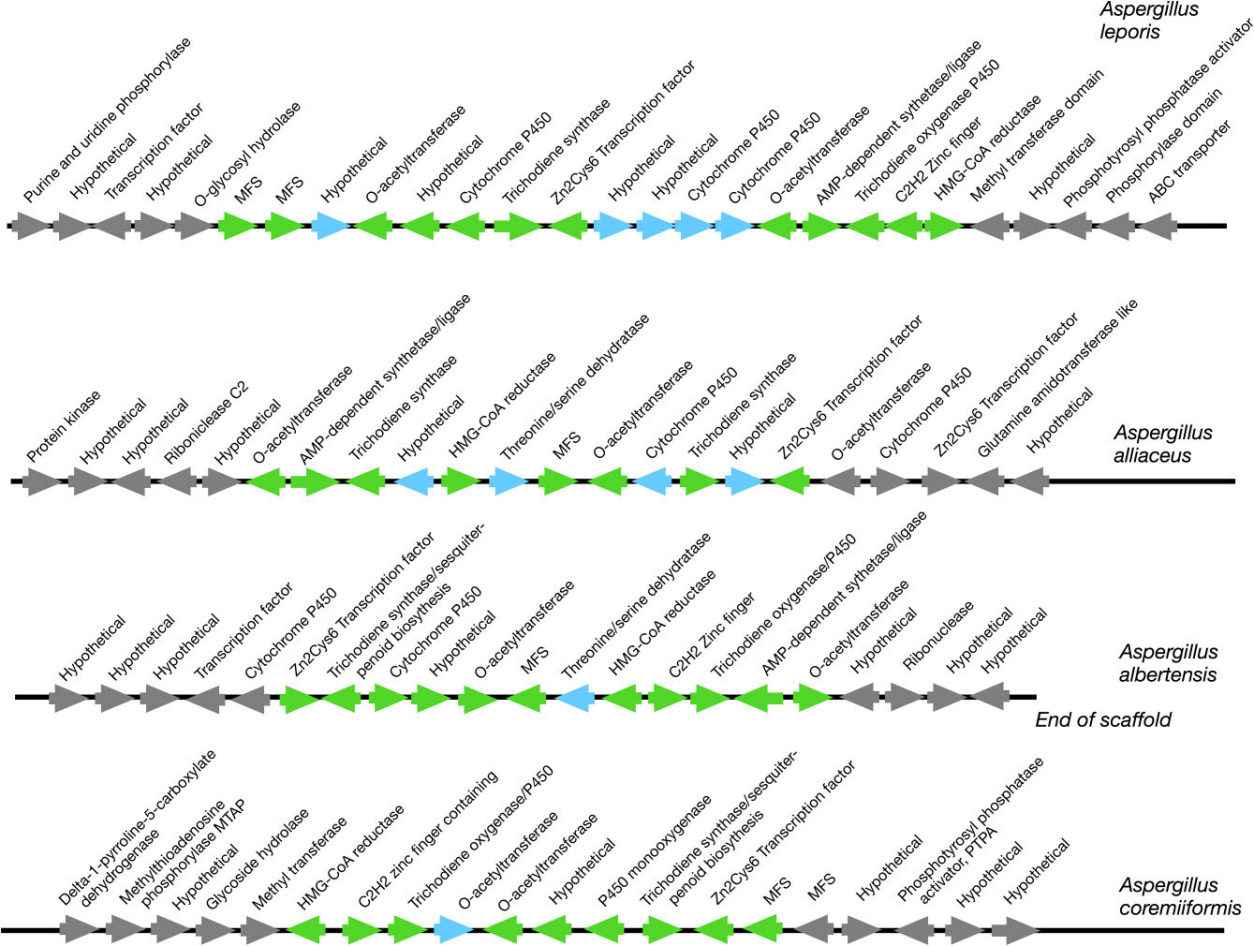
A family of biosynthetic gene clusters with terpene core biosynthetic genes that may produce novel HMG-CoA reductase inhibitors. Green arrows represent genes that are putative BGC members. Blue arrows are genes that are present in some but not all members of the family. Genes in the flanking regions on either side of the BGCs are shown in gray.

Querying our terpene synthase and cyclase databases with a distance of 70 kb returned a total of 26 hits (Supplementary Spreadsheet 2, Terpene Synthase & Cyclase Hits tab). Inspection revealed that several of these were not SM BGCs, and several more BGCs were in the vicinity of additional sterol biosynthesis genes, raising the possibility that they may simply be near a cluster of genes involved in sterol biosynthesis. There was a family of terpene synthase clusters in *Aspergillus leporis* CBS 151.66 v1.0 (Kjærbølling et al., 2020), *Aspergillus alliaceus* CBS 536.65 v1.0 (Kjærbølling et al., 2020), *Aspergillus coremiiformis* CBS 553.77 v1.0 (Kjærbølling et al., 2020), and *Aspergillus albertensis* v1.0 (Kjærbølling et al., 2020) (Supplementary Spreadsheet 2, *A. leporis* Family tab and Fig. 5). They contain genes that putatively encode a Zn2Cys6 TF, an MFS, two O-acetyl transferases, two cytochrome P450’s, HMGCR, a sesquiterpene cyclase, an a C2H2 zinc finger TF, and a conserved hypothetical gene. *Aspergillus. leporis* CBS 151.66 v1.0, *A. alliaceus* CBS 536.65 v1.0, and *A. albertensis* v1.0 contained a gene putatively encoding an AMP-dependent synthetase/ligase. Pairwise BLASTP comparisons revealed that the genes are highly homologous (Supplementary Spreadsheet 2, *A. leporis* Family tab). BLAST searches also revealed that these BGCs are closely related to trichothecene BGCs found in multiple genera of fungi (Proctor et al., 2018, 2020) and, in particular, to the T2 toxin BGC from *Fusarium* species. The trichothecenes share a common core structure but are structurally diverse with approximately 200 structurally distinct trichothecene analogs having been described (Proctor et al., 2020). Trichothecenes are often toxic, but, to our knowledge none have yet been shown to inhibit HMGCR. The regions flanking these BGCs are quite distinct in the four species and, thus, help to define the limits of the BGC.

Finally, querying our DMATS database returned 12 hits (Supplementary Spreadsheet 2, DMAT hits at 70 kb tab. While we found hits that potentially might be BGCs, we found no cluster families.

## Discussion

We have developed a new, computationally light approach toward SM resistance gene-guided genome mining that enables a user to query an entire genomic database for SM BGCs with resistance genes of their choice without having to download any genomes. This allows the user to focus on specific proteins that are promising therapeutic targets. To evaluate this approach, we have searched the genomes in MycoCosm for gene clusters that putatively produce inhibitors of the proteasome β6 subunit and of HMGCR.

With respect to inhibitors of the proteasome β6 subunit, we can draw several conclusions. First, the approach works, revealing known and new fellutamide-family clusters as confirmed by rigorous pairwise BLASTP comparisons. Second, our distance-based approach yielded few hits other than fellutamide clusters. Manual inspection revealed that, when looking at the NRPS clusters and using the 70 kb cutoff, only two of the 81 initial hits were clearly not in partial or complete fellutamide-family clusters. Even when looking at 1825 fungal genomes, our distance-based approach generates very few false positives. Third, there are additional genes within or immediately adjacent to the clusters that could be involved in producing structural variants of fellutamides. It is worth noting that at least four fellutamide variants are known to be produced in nature (Shigemori et al., 1991; Xu et al., 2011), and our data suggest that there could be additional novel fellutamides that potentially have value in medicine or other fields. Finally, we note that our BLAST search returned only 1557 proteasome β6 subunit genes from 1825 genomes. Since each gene should have at least one β6 subunit gene, our list likely did not include some phylogenetically divergent β6 subunit genes. One might be able to obtain a more complete list by using multiple phylogenetically distant β6 genes for BLAST searches.

With respect to HMGCR inhibitor BGCs, our approach was not only effective at finding lovastatin family BGCs, but also revealed previously undocumented BGCs that have a high potential for making HMGCR inhibitors with backbone structures that are different from lovastatin. Rigorous review using pairwise BLASTP comparisons of the initial 85 PKS hits generated by our approach has enabled us to verify, with confidence, 20 PKS hits representing 11 BGCs in 11 genomes (including *A. terreus* NIH 2624) as being surrounded by homologs of lovastatin cluster genes. These are, thus, lovastatin-family BGCs, although the *A. homomorphus* CBS 101889 v1.0 BGC was clearly incomplete and could not make lovastatin. Some of these BGCs (e.g., those in *A. terreus* and *M. ruber*) are known to make lovastatin, but it is possible that others may make analogs of lovastatin such as pravastatin and the various monacolins (Manzoni and Rollini, 2002) or as yet undiscovered lovastatin-related compounds. *Xylaria* sp. FL1777 v1.0, which lacks the *lovF* homolog, cannot make lovastatin but probably makes monacolin J.

More excitingly, our data indicate that there are a substantial number of BGCs that putatively make HMGCR inhibitors with chemical backbones that are different from lovastatin. Some of these BGCs are found only in a single species, as is frequently the case with fungal SM BGCs (de Vries et al., 2017), but we also discovered families of related BGCs. We found three small families of PKS BGCs from eight PKS initial hits that are quite different from lovastatin family BGCs and putatively make novel classes of HMGCR inhibitors (Supplementary Spreadsheet 2, *A. olivicola* Family, *A. transmontanensis* Family, and *A. japonicus* Family tabs). In addition to these PKS clusters, we found one interesting terpene cluster family (Supplementary Spreadsheet 2, *A. leporis* Family tab), and, even more excitingly, we found a very interesting family of 19 NRPS clusters which are in six genera of basidiomycetes (Supplementary Spreadsheet 2, Pisolithus Family tab)—exemplifying that our simple distance-based approach can find resistant SM BGCs across the entire fungal kingdom. The products of each of these BGCs are predicted to be HMGCR inhibitors, and it is possible that some may be more medically efficacious than lovastatin or amenable to chemical modification to make particularly efficacious HMGCR inhibitors. It is also possible that some of them might have different specificities from lovastatin. One can speculate, for example, that some of them might specifically inhibit fungal HMGCRs since other fungi are among the the chief competitors of these organisms.

Our results with HMGCR and the proteasome β6 subunit as targets are of intrinsic interest that could, potentially, lead to the development of new therapeutics. Just as importantly, they validate our approach and suggest that this approach can be used to identify BGCs that produce compounds that inhibit other targets of choice.

### Effects of Altering the Distance Cutoff in Our Script

One might worry with a distance-based approach that increasing the distance threshold would result in false positives in which the putative resistance gene is actually a housekeeping gene that happens to be near a BGC. Our data indicate, however, that this is not a significant concern. Our most informative data are from our search for β6 proteasome subunit inhibitors. Our NRPS search querying 1825 genomes with a 70 kb threshold returned 81 initial hits, and in all but two cases (*A. multicolor* v1.0 and *Amylomyces rouxii* NRRL 5866 v1.0) the β6 subunit was associated with a fellutamide-family BGC. Supplementary Fig. S5 is a histogram plotting the frequencies of hits as a function of distance between the centers of the NRPSs and the proteasome subunits, out to a cutoff of 100 kb. In 77/84 cases the NRPS and proteasome subunit were less than 20 kb apart. Extending the distance cutoff from 70 to 100 kb added only three additional hits. Examining these hits in MycoCosm revealed them to be false positives. Querying our PKS, terpene synthase, terpene cyclase, and DMATS databases with a distance cutoff of 70 kb returned only 10 hits, nine of which were likely false positives, with one uncertain.

With respect to putative HMGCR inhibitor BGC hits across all SM backbones, the majority are new, and they have not yet been experimentally validated. Except for the lovastatin-family BGCs, we cannot say, with confidence, whether these hits are valid or false. In the great majority of cases, however, the putative HMGCR resistance gene appears to be associated with an SM BGC (based on the predicted functions of the proteins encoded by nearby genes), suggesting that the hits are valid. Both β6 proteasome subunit and HMGCR searches used a generous 70 kb cutoff that is unlikely to exclude any valid hits, and they resulted in a small and easily manageable number of likely false positives. While reducing the cutoff would reduce the number of false positives, the benefit is small when weighed against the possibility of missing potentially valuable BGCs.

### Comparisons With Other Approaches

FRIGG (fungal resistance gene-directed genome pipeline) (Kjærbølling et al., 2019b) is designed to work with whole genome sequencing data. It identifies SM BGCs and defines their boundaries based on a fungus-optimized version of the SMURF SM BGC prediction algorithm (Khaldi et al., 2010). It then determines if a member of a list of putative essential resistance gene is found within these clusters, while applying several filtering steps. The authors defined their list of putative essential genes by looking for genes that were conserved in >90% of the organisms under study. It should be pointed out, however, that some conserved genes are not essential. For example, the conserved cyclin gene *clbA* is not essential in *A. nidulans* (Paolillo et al., 2018), nor are the conserved γ-tubulin complex genes *gcpD, gcpE*, and *gcpF* (Xiong and Oakley, 2009) or the histone H1 gene *hhoA* (Ramón et al., 2000). This approach depends on SM BGCs being accurately predicted by SM cluster prediction algorithms, and this is not always the case (Inglis et al., 2013). In spite of these caveats, FRIGG has a big advantage in that it can potentially reveal new target proteins.

Our results highlight a particular problem in predicting clusters that harbor a resistant allele of an essential gene. Since the resistant allele has no apparent SM function, it may cause the boundary of the cluster to be called incorrectly, resulting in false negatives due to the resistant allele being excluded from the BGC. Among the 39 complete fellutamide family BGC we found, SMURF did not predict the β6 proteasome subunit gene to be a member of the BGC in seven cases (all five of the *Lobaria* isolates plus *Aspergillus indicus* v2.0, and *T. paradoxa* CBS 103.73 v1.0). Among the 10 complete lovastatin family clusters, and SMURF did not predict the HMGCR gene to be a member of the BGC in three cases (*C. orchidophilum* IMI 309357, *M. ruber* NRRL 1597 v1.0, and *A. chrysellus* CBS 472.65 v1.0). A resistance gene-guided genome mining approach using the SMURF cluster prediction algorithm would have missed these fellutamide and lovastatin clusters entirely.

Liu *et al.* (2021) have developed a resistance gene-guided genome mining algorithm for use with MATLAB to identify BGCs that encode CYP51 inhibitors. The approach is similar to ours in that is uses distances between the core biosynthetic and resistance genes, but it differs in important ways. It requires the user to download all the genomes being studied and is built to be used with unannotated genome sequences. It also deals with target genes differently, only including genomes that have more target genes than the average number in all the analyzed genomes. Additionally, it imposes additional filtration constraints based on homology score, and requires specific gene colocalizations.

FunARTS (Fungal bioActive compound Resistant Target Seeker) (Yilmaz et al., 2023) is a recently published “exploration engine” for resistance gene-guided genome mining. It requires the user to download genome sequences which are then analyzed using Hidden Markov Models to identify “core genes” which are putative essential housekeeping genes. SM BGCs are identified using fungiSMASH a fungal optimized version of antiSMASH (Blin et al., 2021). Core genes can be analyzed to determine if there are duplicates in the genome and they can be filtered for proximity to BGCs. FunARTS is quite flexible, particularly with respect to outputs, and it can, in principle, find new resistance genes. One weakness of FunARTS is that fungiSMASH may not call BGC boundaries correctly. Another, that the authors point out, is that the current procedure for identifying core genes is imperfect. For example, there were no FunARTS hits for four notable resistance genes including HMGCR and the important antifungal target β-1,3-D-glucan synthase because they were not identified as core genes by the Hidden Markov Model employed in FunARTS. Although these are acknowledged weaknesses, FunARTS is certainly a very valuable tool, and it has the notable strength that it can identify novel gene targets whereas our approach cannot.

Significant advances have also been made in resistance gene-guided genome mining in bacteria. In fact, Antibiotic Resistant Target Seeker (ARTS) (Alanjary et al., 2017) and its more advanced successor ARTS 2.0 (Mungan et al. 2020) are predecessors of FunARTS. The approach of ARTS and ARTS 2.0 is similar to that of FunARTS, with similar strengths and limitations, but ARTS was focused on actinomycetes whereas ARTS 2.0 is focused on the entire bacterial kingdom.

The workflow for our approach is quite different from those of FRIGG, FunARTS and the approach of Liu et al. (2021). The FRIGG and Liu et al. approaches require the downloading of genomes that one wants to analyze. FRIGG returns both target molecules and the BGCs that putatively make inhibitors of the target molecules. The Liu et al. approach works for a single target molecule at a time. With FunARTS one uploads a genome or genomes of interest to a server that then analyzes it in a variety of ways specified to some extent by the user before outputting interactive summary tables. Our approach works with genomes that have already been sequenced and annotated. The user interacts with the database to create files of core biosynthetic SM genes. These can be used repeatedly for searches with different target molecules. Comma separated values files for new target molecules can be generated through blast searches. Our Python script analyzes the files very rapidly and returns hits that are then analyzed with tools chosen by the user. The script requires only seconds to run even with large target and core SM biosynthetic gene files. We typically use a MacIntosh computer running Python in Xcode, but most desktops and laptops will be more than adequate.

### Implications With Respect to Horizontal Transfer of Secondary Metabolism Gene Clusters

In our search for proteasome β6 inhibitors, we found only fellutamide-family BGCs, but we found them in 41 genomes from species that are in three different classes of fungi, the Leotiomycetes, Eurotiomycetes, and Lecanoromycetes. The fact that we have found closely related clusters among members of phylogenetically distant organisms strongly suggests the clusters have been transferred horizontally. If the fellutamide clusters were exclusively inherited vertically, one would expect that they would fall into phylogenetically related phyla and be common in those phyla. Within the Eurotiomycetes, there appears to be both horizontal and vertical gene transfer. We found fellutamide clusters in 33 *Aspergillus* genomes and in *T. paradoxa* CBS 103.73 v1.0, which is phylogenetically distant, suggesting that *T. paradoxa* CBS 103.73 v1.0 acquired a fellutamide BGC through a horizontal gene transfer event. All of the *Aspergillus* genomes with fellutamide-family clusters, however, are members of species that are in sections Aeni and Nidulantes of subgenus Nidulantes (Chen et al., 2016). No other *Aspergillus* species had fellutamide clusters, and this strongly suggests that the fellutamide cluster was vertically inherited from a common ancestor of modern-day Aeni and Nidulantes section Aspergilli. We found lovastatin family clusters in 11 genomes from species that are in two separate classes, the Eurotiomycetes and Sordariomycetes, suggesting that lovastatin family clusters have been transferred horizontally.

## Conclusion

We have developed a new, computerized approach to resistance gene-guided genome mining. We have tested it by searching the genomes in MycoCosm for BGCs that are predicted to produce inhibitors of the proteasome β6 subunit and HMGCR. We have found known examples of fellutamide and lovastatin clusters as well as previously undiscovered but related BGCs. With respect to BGCs that putatively target HMGCR, moreover, we have found new families of BGCs that are predicted to produce HMGCR inhibitors with chemical backbones that are different from lovastatin. It should be possible to express these BGCs to identify their products and determine if they have medical or agricultural value. Finally, this approach can be used to look for BGCs that produce inhibitors of additional target molecules that may have anti-fungal, antibiotic, or other useful properties.

## Supplementary Material

kuad045_Supplemental_Files

## Data Availability

All data are present in the manuscript, supplementary data or the databases cited (principally MycoCosm).
