# Supplementary material for: Computer-aided, resistance gene-guided genome mining for proteasome and HMG-CoA reductase inhibitors
Source: J Ind Microbiol Biotechnol. 2023 Dec 7;50(1):kuad045. doi: 10.1093/jimb/kuad045 (PMC10734572; doi:10.1093/jimb/kuad045)
Supplement: kuad045_Supplemental_Files [file kuad045_supplemental_files.zip › Jenkinson et al Supplementary Figures.pdf]

Figure S1

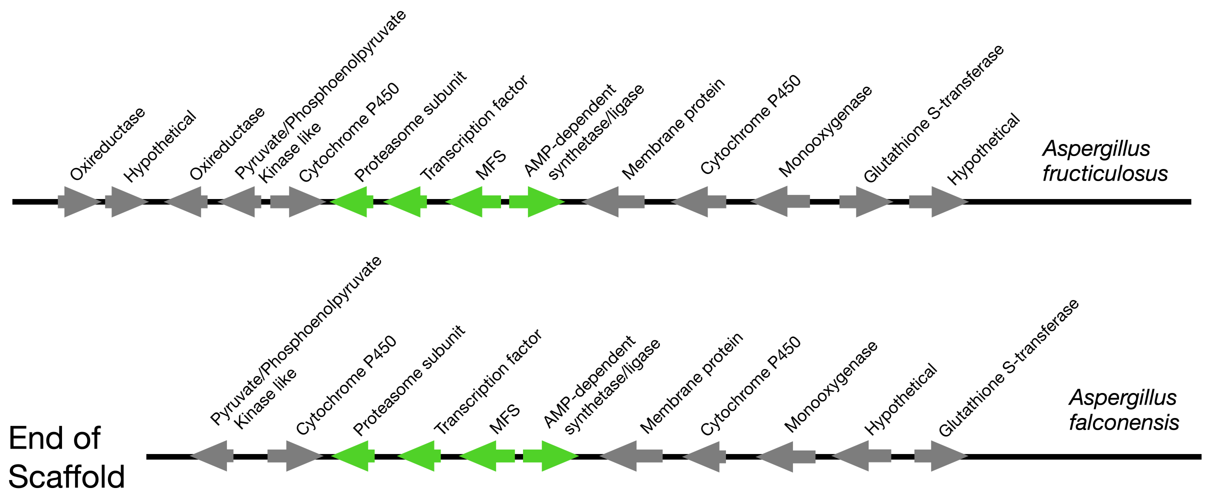

Figure S1. Fellutamide-related clusters in *A. fructiculosus* v1.0 and *A. falconensis* CBS 271.91 v1.0. Genes with a high degree of similarity to fellutamide BGCs in other fungi are shown in green. Percentage identities are shown in Supplementary Spreadsheet S1, Fellutamide Cluster BLASTPs tab. The order and directions of transcription for the genes are shown but the gene sizes and distance between genes are not drawn to scale. The *A. falconensis* scaffold terminated at the left as shown while the *A. fructiculosus* scaffold terminated at the right shortly beyond the region shown. The regions are highly syntenic.

Figure S2

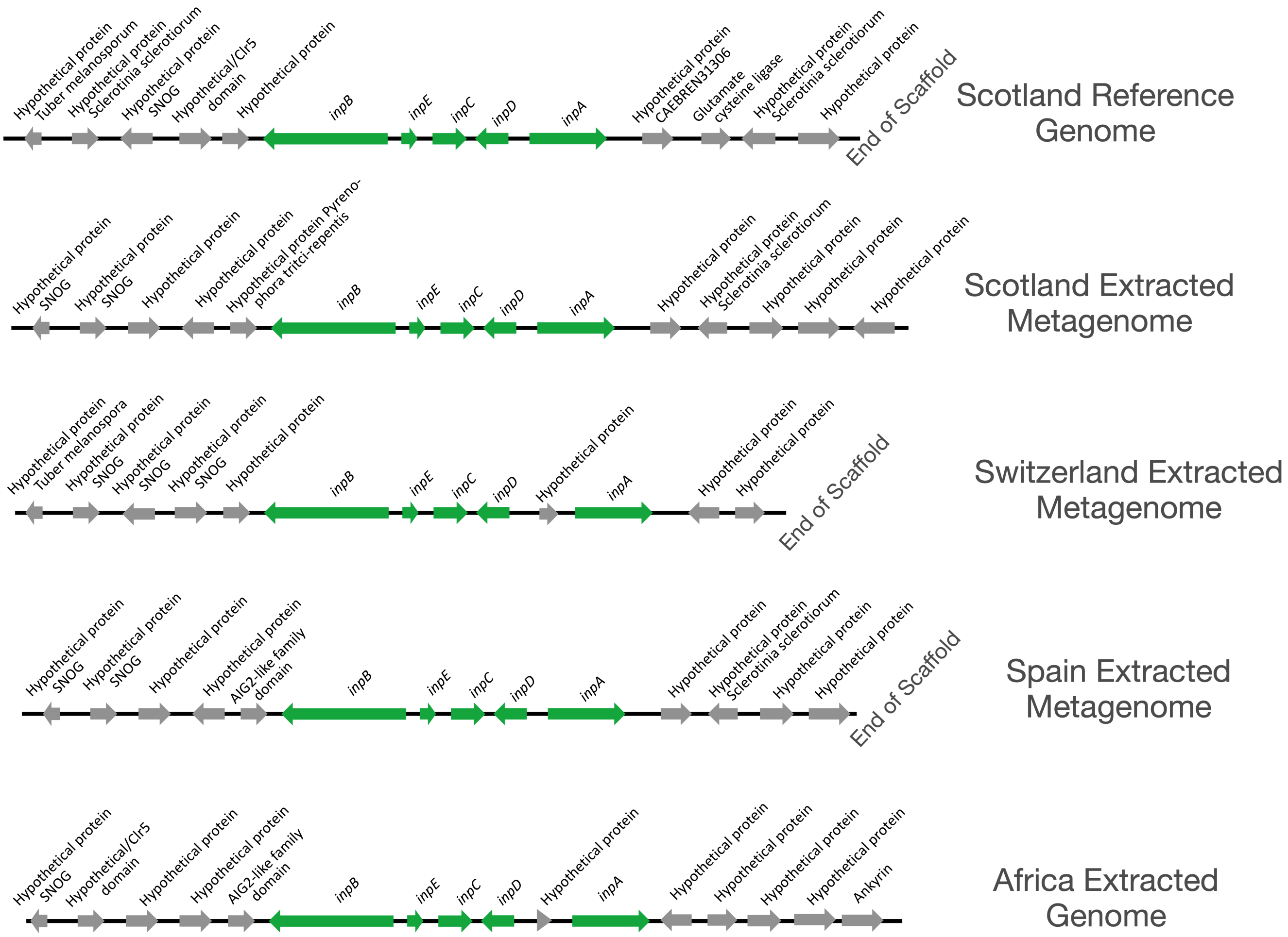

Figure S2. Fellutamide family biosynthetic gene clusters in *Lobaria pulmonaria.* The fellutamide BGCs are shown in green. The two hypothetical protein genes in the Switzerland metagenome and the Africa genome clusters are small open reading frames and are likely annotation artifacts. While there are similar genes in the regions flanking the fellutamide BGCs, they are not identical.

Figure S3

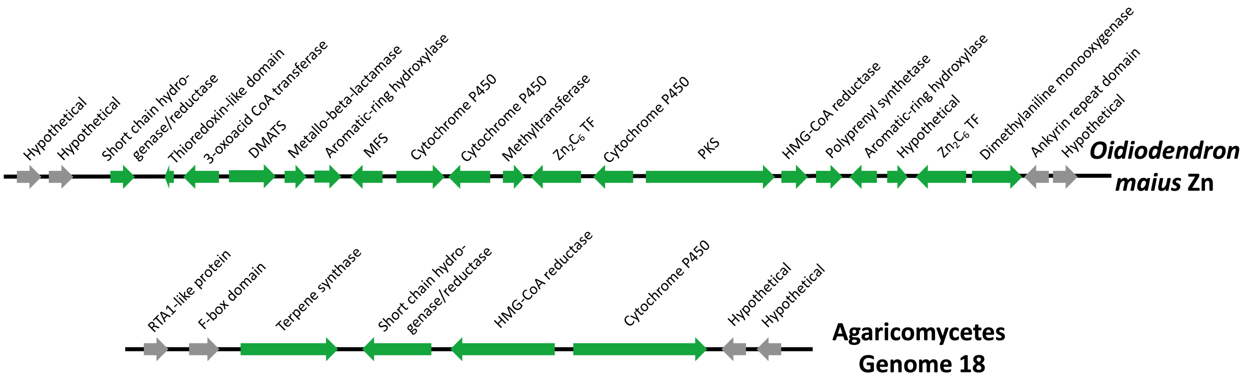

Figure S3. Two unique BGCs that may produce HMG-CoA reductase inhibitors. The *Oidiodendron maius* BGC contains an HMGCR gene and a number of genes with predicted functions in secondary metabolism including a PKS, a potential core biosynthetic gene. The core biosynthetic gene for the Agaricomycetes BGC is a terpene synthase.

Figure S4

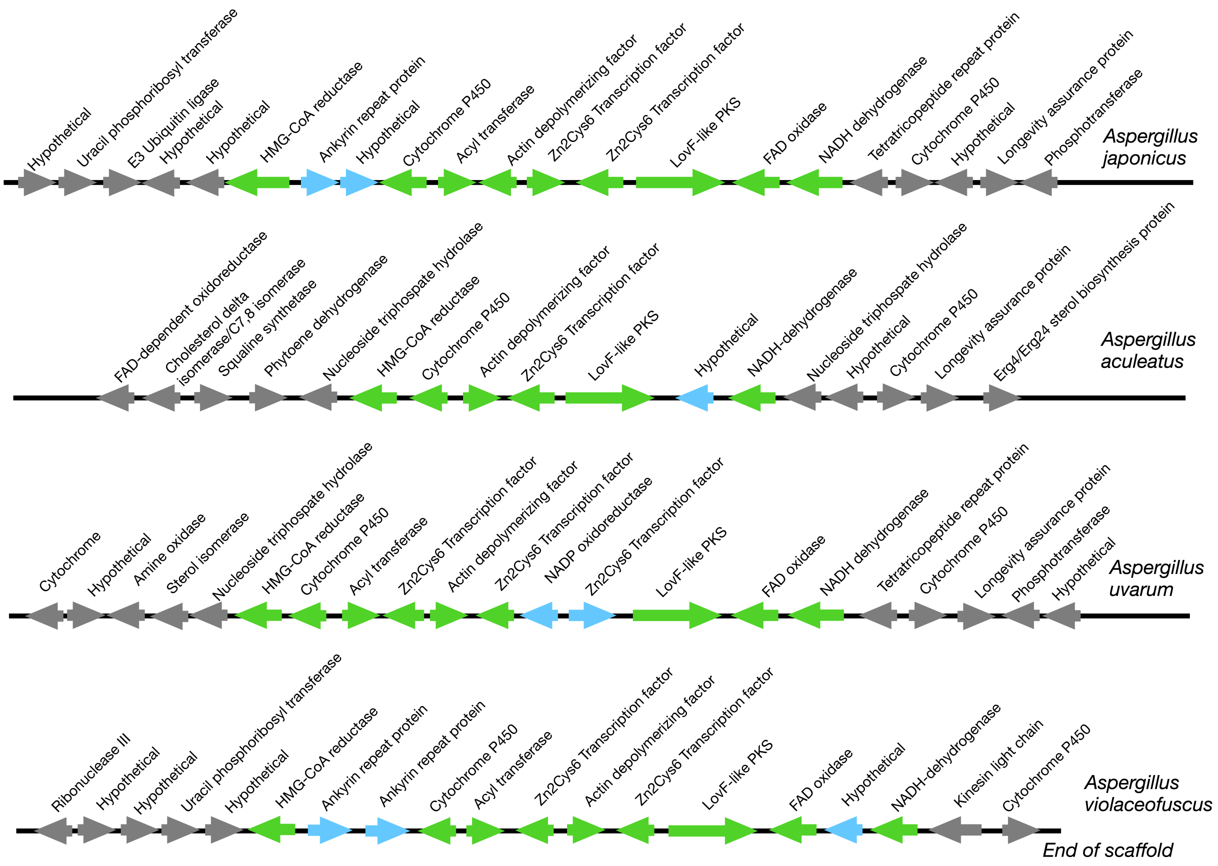

Figure S4. A non-lovastatin family of BGCs that may produce novel HMG-CoA reductase inhibitors. Green arrows represent genes that are likely BGC members. They are highly conserved among all the family members (Supplemental Spreadsheet 2, A. japonicus family tab). Blue arrows represent genes found in a subset of the BGCs and gray arrows are genes that flank the BGCs. The BGCs are clearly related but appear to have changed over time. Five flanking genes are shown at each end of each BGC except for the right flank for *A. violaceofuscus* in which the scaffold ends after two genes. Although some homologs are shared, the flanking regions are distinct.

Figure S5

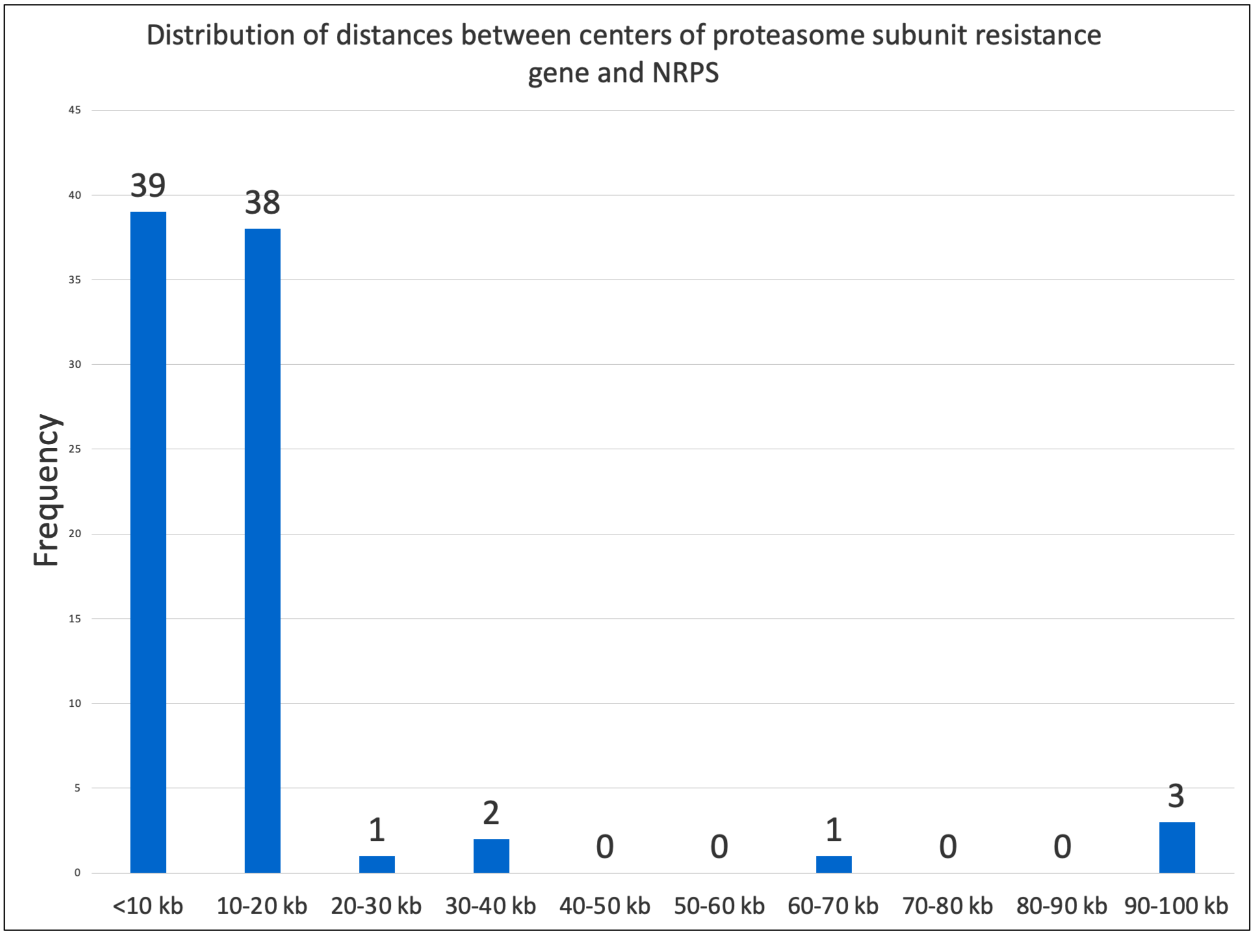

Figure S5. Distribution of distances between the centers of proteasomal resistance genes and NRPSs out to 100 kb. In 77/84 hits the center-to-center distances between the resistance genes and NRPSs was less than 20 kb. The hits in the 60-70 kb and 90-100 kb bins were artifactual. If a cutoff of 40 kb had been used, no genuine fellutamide BGCs would have missed. On the other hand, using an unreasonably large cutoff such as 100 kb would only give a few false positive hits.
